# The Evolution Road of Seaweed Aquaculture: Cultivation Technologies and the Industry 4.0

**DOI:** 10.3390/ijerph17186528

**Published:** 2020-09-08

**Authors:** Sara García-Poza, Adriana Leandro, Carla Cotas, João Cotas, João C. Marques, Leonel Pereira, Ana M. M. Gonçalves

**Affiliations:** 1 Department of Life Sciences, Marine and Environmental Sciences Centre (MARE), University of Coimbra, 3000-456 Coimbra, Portugal; sara.poza13@gmail.com (S.G.-P.); adrianaleandro94@hotmail.com (A.L.); jcotas@gmail.com (J.C.); jcmimar@ci.uc.pt (J.C.M.); leonel.pereira@uc.pt (L.P.); 2 LEPABE—Laboratory for Process Engineering, Environment, Biotechnology and Energy, Faculty of Engineering, University of Porto, 4200-465 Porto, Portugal; carlacotas@gmail.com; 3 Department of Biology and CESAM, University of Aveiro, 3810-193 Aveiro, Portugal

**Keywords:** seaweed, healthy benefits, aquaculture, offshore, onshore, IMTA, compounds, industry 4.0

## Abstract

Seaweeds (marine macroalgae) are autotrophic organisms capable of producing many compounds of interest. For a long time, seaweeds have been seen as a great nutritional resource, primarily in Asian countries to later gain importance in Europe and South America, as well as in North America and Australia. It has been reported that edible seaweeds are rich in proteins, lipids and dietary fibers. Moreover, they have plenty of bioactive molecules that can be applied in nutraceutical, pharmaceutical and cosmetic areas. There are historical registers of harvest and cultivation of seaweeds but with the increment of the studies of seaweeds and their valuable compounds, their aquaculture has increased. The methodology of cultivation varies from onshore to offshore. Seaweeds can also be part of integrated multi-trophic aquaculture (IMTA), which has great opportunities but is also very challenging to the farmers. This multidisciplinary field applied to the seaweed aquaculture is very promising to improve the methods and techniques; this area is developed under the denominated industry 4.0.

## 1. Introduction

Seaweeds are benthic organisms ubiquitously distributed along coasts from tropical to polar regions. They are part of Plantae kingdom, and, as land plants, seaweeds also constitute the basis of the food chain but in aquatic ecosystems [1]. Among the major primary producers, seaweeds or benthic marine algae grow in the intertidal and sub-tidal regions of the sea and contain photosynthetic pigments, which lead them to photosynthesize and produce food.

Seaweeds are grouped in three divisions: brown algae (Ochrophyta-Phaeophyceae), red algae (Rhodophyta) and green algae (Chlorophyta). These organisms are producers of many structural molecules (primary metabolites), such as proteins, lipids and carbohydrates, and they also produce other interesting bioactive compounds (secondary metabolites) that can have applications in many sectors (food, feed, agriculture, cosmetics, pharmaceutical andbiotechnological) [2].

Since elder times, seaweeds have been used as food in some civilizations around the world [3]. Furthermore, it has been reported that edible seaweeds are rich in proteins, lipids and dietary fibers [4,5,6]. The high levels of minerals and dietary fibers, as well as low lipid levels that characterize many seaweed species, make marine algae an attractive raw material for supplying bioactive substances with a wide range of applications [5,6]. In addition, the quality of their proteins [5,7,8] and antioxidant activities, associated with their content of polyphenolic compounds [9] and pigments (e.g., fucoxanthin [10]) turn seaweeds into an interesting source of bioactive substances used especially in human and animal nutrition. Seaweeds also contain high quantities of vitamins (A, K andB12), protective pigments, minerals and trace elements that are essential for the human diet and may collaborate with many EU-approved nutritional claims (such as iron, calcium, iodine or magnesium) relative to bone health, cognitive function, maintenance of normal metabolism, normal growth and muscle function, among others [6,11,12,13,14]. Polyunsaturated fatty acids (PUFAs), mainly omega 3 (ω-3) and omega (ω-6), are the principal components of their cell membranes, so seaweed can also be a source of essential fatty acids [15,16].

Many investigations demonstrated the nutraceutical, pharmaceutical and cosmeceutical value of the seaweeds. Some of their diverse properties are anti-cancer, antiviral, antifungal, antidiabetic, antihypertensive, immuno-modulatory, cytotoxic antibiotic, anticoagulant, anti-inflammatory, anti-parasitic, antioxidant, UV-protective and neuroprotective [2,13,17,18,19,20,21,22,23]. It has also been confirmed that several species of seaweed have powerful antioxidant compounds such as phlorotannines, carotenoids and sterols, making seaweed a source of compounds with possible neuroprotective effects, useful in the treatment of neurodegenerative diseases such as Parkinson’s and Alzheimer’s [24,25]. Sulfated polysaccharides from seaweed have shown important potential pharmacological uses, such as their anti-ulcer effects, by preventing adhesion of the infection caused by the bacteria Helicobacter pylori [26].

These marine organisms are normally used in the cosmetics sector as bioactive extracts, coloring agents, texturing stabilizers or emulsifiers and are a source of different compounds used in skincare [27]. Due to seaweeds being photosynthetic organisms, they generate compounds that absorb UV rays, such as carotenoids and terpenes, mycosporin-like amino acids (MAAs) and phenolic compounds, which are useful photo-protective elements for the formulation of sunscreens [28].

Thus, due to all these bioactivities and potential novel applications, seaweeds have been showcased as a sustainable resource for the future, which is leading to an increased demand of these organisms’ exploitation and consequently also in their production. Moreover, the biological productivity of the seaweed causes photosynthetic carbon storage. This carbon can be immobilized in sediments or moved to the depths of the sea resulting in a CO_2_ sink. Thus, collecting algae and using them to produce biofuels and in other industries (food, feed, pharmaceuticals and fertilizers) can help in CO_2_ mitigation [29]. Seaweed can be used as carbon trap and then as fuel [29,30] and can provide a sustainable alternative source of biomass for the fuel production and also for chemicals, such as bioethanoland bio-butanol [31,32,33,34]. Furthermore, high levels of dissolved inorganic nutrients, such as nitrogen, phosphorous and carbon, are taken up by seaweed leading to the algal growth and helping to alleviate eutrophication in seas and oceans [2,35].

Several seaweeds are structuring species in coastal zones, changing the environment (by modifying light, sedimentation rates and hydrodynamics) [36,37,38,39]. Seaweeds are part of food webs and give ecosystem services such as habitats, food and refuge to a diversity of associated organisms (which are of conservation and economic importance) from different trophic levels (apex predators, fishes and invertebrates) [40,41] and therefore support biodiversity [42]. In addition, marine seaweeds contribute to the coastal defense by reducing the hydrodynamic energy from waves and by maintaining a high bed-level at tidal flats, thus protecting those tidal areas from erosion [43,44].

The demand for seaweeds and their products has been growing globally and so has the interest in their production and the attraction of stakeholders to invest more widely in the production of various algal species that may fill different economic sector needs [45]. This is extremely important to suppress the need to feed a growing population, on a planet where there will not be enough land for agricultural crops, as seaweed production does not compete for inland arable land, freshwater or agriculture fertilizers [46,47,48,49]. However, it does compete with other near-shore activities such as saliculture, fish and invertebrate’s aquaculture or even agriculture. Fertilizers are only used in inland cultivation system, although they present a low percentage of usage in aquaculture, being seawater rich in nutrients from other species’ aquacultures normally used [46,47,48,49].

Thus, seaweed aquaculture offers a variety of opportunities to mitigate and adapt to climate change and support biodiversity. However, there may be some negative impacts, such as the unintentional introduction of non-indigenous “hitchhiker” species, including pathogens [50].

To conclude, this review aims to provide an overview on seaweed aquaculture, gathering the recent developments, with emphasis on new methods to potentiate the production of compounds of interest to different sectors, from biotechnology to pharmaceutical and nutraceutical [2].

## 2. Seaweeds Biodiversity and Potential to Exploitation

The principal phyla of seaweed are Chlorophyta (green algae), Ochrophyta-Phaeophyceae (brown algae) and Rhodophyta (red algae). Each phylum is composed of thousands of species [51]. Food, folk remedies, dyes and fertilizers traditionally use seaweed in their confection. In the early 1900s, seaweed components were launched industrially due to the development of mass food production [52].

In the nutraceutical, pharmaceutical and biotechnological industries, there are some applications to hydrocolloids, for instance alginate, carrageenan and agar are used due to their gelling features [53,54]. However, other minor components of the seaweeds, as will be presented later in this review, could be applied in high-value products, making seaweed aquaculture even more profitable for the seaweed producers [55,56,57,58]. During the past thirty years, enthusiasm has grown in seaweed as functional foods (nutraceuticals), enabling dietary advantages superior to their macronutrient content. Furthermore, to produce therapeutic products, seaweeds have been targeted for the obtention of metabolites with biological activity [55,58].

Despite all research studies performed in this field to demonstrate the bioactivities of seaweed-derived compounds, there is not the same expression in effective products on the market [56,57]. Consequently, more research and standardized assays need to be done, where the main questions are the compound bioavailability in seaweed, the low efficiency and efficacy of the extraction and the isolation and characterization of the biomolecules [59,60,61]. Some compounds could be difficult to isolate due to their biochemical features (e.g., size, molecular weight, structural similarities or even the tendency to bind or react with other molecules) [62].

However, seaweeds are viewed as promising functional foods and as food supplements [63,64], where the lower heavy metals concentration safeguard needs to be assured. Nevertheless, there is a need for more research to clarify the seaweed state, such as their role in nutrition and disease prevention [18]. However, there are various seaweeds’ compounds commercially available, where the seaweeds’ polysaccharides represent a large portion of that market, used for various industries, such as food and pharmaceutical [65,66,67,68]. The seaweed polysaccharides are considered dietary fibers, although assay with vegetal jelly (carrageenan) has proven to reduce cholesterol [69,70]. In the case of proteins, the research is ambiguous regarding the digestibility, due to the interaction of the proteins with other compounds [69]. They present a low concentration of lipids, despite the amount of ω-6 and ω-3 [71,72]. Moreover, the seaweed mineral content is the most important because minerals are essential for the human cells to work properly [3,63,64,73,74].

Nevertheless, there is the need to execute further in vivo and clinical studies to guarantee that the selected raw materials maintain the great potential and are safe, as well as to perform accurate controls throughout all the production phases of industrial batches [75].

### 2.1. Green Seaweeds

The green seaweeds (Chlorophyta) are green since no other pigments mask the chlorophyll. In fact, these seaweeds have chlorophylls (a and b) and carotenoids (β-carotene and xanthophylls), that are important in the protection against harmful effects experienced due to irradiance [76], having an antioxidant activity [77].

In terms of polyunsaturated fatty acids (PUFA), Chlorophyta are mostly composed by the C16 and C18 PUFA, namely the Linoleic acid (LA; C18:2ω-6) in most of the species. However, α-linolenic acid (ALA, C18:3ω-3) is characteristic of Ulvales [16,78,79,80,81,82]. Contrasting with red and brown algae, green algae also contain large amounts of Palmitolinolenic (16:3ω3) and Palmitidonic (16:4ω-3) PUFAs. Regarding the carbohydrates, Chlorophyta are rich in sulfated polysaccharides that constitute the cell walls [83]. Particularly from the Ulvaceae, water-soluble molecules, ulvans, could be obtained.

Ulvans are characterized as a sulfated single polydisperse heteropolysaccharide composed of variable quantities of uronic acids, including glucuronic and iduronic acids alternating with neutral sugar moieties, for example rhamnose, xylose and glucose, connected by α- and β-1→4 bonds [83,84]. Ulvans account for 18–29% of the carbohydrate fraction of green algae [85] and their bioactivities vary depending on the structural aspects of the molecule in question (e.g., molecular weight, degree or pattern of sulfatation, sugar constitution, linkages, isomers, and degree of branching). Thus, obviously, when obtained from different species of *Ulva*, and specimens from different environments, they exhibit diverse bioactivities [86]. They are of biomedical interest, namely for applications in tissue engineering, biofilm prevention and drug delivery once it was proven that ulvans can be recognized by hepatocyte membrane receptors [83,84,87,88,89]. These compounds have antiviral, antioxidant, anticoagulant, antihyperlipidemic and anticancer activity, in addition to immunostimulatory effects [32,83,84].

Ulvans are a high-value product in themselves, with unique gelling, bioactive and functional properties [83,90]. Moreover, it has been reported that this anionic polysaccharide gives *Ulva* sp. the ability to accumulate heavy metals, removing them from contaminated waters to the seaweed tissue where it is not available until the seaweed is destroyed [91,92,93]. This makes the *Ulva* spp. particularly suitable to mitigate impacts from anthropogenic wastewaters because of their high productivity and resilience to diverse growing conditions [32,94,95,96]. This seaweed used in heavy metal bioremediation needs to be carefully used, and there is research to use seaweed to remove heavy metals for their recuperation, promoting a heavy metal circular economy [97,98]. In the agriculture field, ulvans improve plant immune responses [84,99].

Within green algae, such as *Codium*, *Ulva* and *Chaetomorpha* spp., there are also other compounds of interest, e.g., sterols. These genera are especially rich in 28-isofucosterol [79,100] and also in ergosterol and 24-ethylcholesterol [4].

Thus, compounds extracted from green seaweed are very versatile and could be applied as pharmaceuticals, nutraceuticals, functional foods and feed, in agriculture and bioremediation.

The compounds extracted are bioavailable for humans, mainly the PUFAs, however, the ulvans are not digestible by humans, although they serve as a dietary fiber. *Ulva compressa* extracts are used for cosmetics, derivate from various biological activities [101].

### 2.2. Brown Seaweeds

The predominance of fucoxanthin characterizes the brown seaweeds (Phaeophyceae), that is, along with the chlorophylls, a pigment of this algae group [24,77,102,103,104]. Fucoxanthin contains an anallenic bond and a 5,6-monoepoxide. Different brown seaweed strains produce different compositions and profile of fucoxanthin [24]. Studies showed that fucoxanthin has anti-tumoral, antioxidant and anti-obesity properties [105,106,107,108].

In the fatty acids content, the most abundant saturated fatty acids (SFA) are myristic (C14:0) and palmitic (C16:0) acids [16,81,109,110,111,112]. Regarding the PUFAs, brown seaweeds are mainly constituted of Linoleic acid (LA, C18:2ω-6), arachidonic acid (AA, C20:4ω-6) and Eicosapentaenoic acid (EPA, C22:5 ω-3) [81,109,113]. Cholesterol is one of the major sterols presented in all groups of seaweed [114,115]. Besides that, brown and green algae are rich in other C29 sterols, particularly fucosterol and isofucosterol, respectively [116,117,118].

The phenolic compounds most present in brown algae are meroditerpenoids (plastoquinones, chromanolsand chromenes), which are found almost exclusively in the Sargassaceae [119]. The phenols have been demonstrated to have anti-diabetic [120,121], anti-HIV, anticancer, bactericidal, antiadipogenic, antiallergic and neuroprotective effects, among other biological activities [122,123,124,125,126]. These phenolic compounds can interfere in the amino acid bioavailability when the seaweed is consumed, although these compounds are considered the seaweed-flavors, due to the impact in flavors of the seaweeds and in the fish [127,128]. Thus, there are aquatic feeds with seaweeds’ phenolic to provide “oceanic flavor” to the fish farmed in-land. In addition, the phlorotannins are being used as antidiabetic, anti-obesity, bone regeneration and for cardiovascular diseases, mainly dieckol extracted from cultivated *Ecklonia cava* [121,129,130].

In terms of polysaccharides in brown seaweeds, the most specific one is the alginate or alginic acid, in which appear 1,4-linked β- d-mannuronic and α- l-guluronic acid residues organized in a non-regular blockwise order across the chain [131]. Alginates are found in the cell walls of brown seaweed and have different chemical structures and characteristics, according to different genera of brown seaweed. *Ascophyllum, Durvillaea*, *Ecklonia*, *Laminaria*, *Lessonia*, *Macrocystis* and *Sargassum* spp. are some of the species of brown seaweed that contain alginate [132]. A source of alginates is also found in *Ecklonia radiata* which belongs to the same brown algal order (Laminariales, also admitted as kelps) as *Saccharina japonica* and *Undaria pinnatifida*, mainly grown for human consumption [133,134]. Fucales (large brown seaweed) also utilized for nutrition and alginates, include *Scytothalia dorycarpa* (family Seirococcaceae), *Cystophora subfarcinata* and *Sargassum linearifolium* (both Sargassaceae) [133]. *Laminaria hyperborea*, *Laminaria digitata*, *Saccharina japonica*, *Ascophyllum nodosum*, *Ecklonia maxima*, *Macrocystis pyrifera*, *Durvillea antarctica*, *Lessonia nigrescens* and *Lessonia trabeculata* are the brown algae most commonly employed for the manufacture of alginate, normally picked from the sea or acquired from the shore [135].

Alginates in brown seaweed can impose a difficulty to the availability of the protein molecules due to their high viscosity and anionic cell-wall polysaccharides which may affect the success of the extraction of algal proteins [136]. Alginate can help reducing blood levels of cholesterol and glucose because it helps to develop intestinal viscosity, due to being a soluble dietary fiber [137,138].

Alginates are used in food, cosmetic, textile, construction and pharmaceutical/biomedical industries due to their ability to be used as emulsifiers, thickeners, binding and gel-forming agents because of their capability to condense aqueous solutions and assembling gels [132]. Another polysaccharide is the Laminarin which is the main storage polysaccharide of *Laminaria* spp. (over 36% of the dry weight depending on the season). It is a short polymer of about 20–25 glucose residues linked by β(1–3) bonds with some β(1–6) bonds that lead to a ramification of the molecule [139,140,141]. The composition of laminarin is also modified by other environmental causes such as water temperature, salinity, waves, sea current and depth of immersion and these factors influence its bio-functional activity [142].

The content of laminarin from brown algae is over levels of 35% on a dry basis, which changes with the species, harvesting season, habitat and method of extraction [141]. The principal source of laminarin and the laminarin content of several usually used seaweed are: *Saccharina latissima* 0–33% of dry weight, *Laminaria hyperborean* 0–32% of dry weight [69], *Laminaria digitata* 14% of dry weight [6], *L. digitata* 0–35% of dry weight based on season [143], *Fucus vesiculosus* 84% of total sugars [142], *Undaria pinnatifida* 3% of dry weight and *Ascophyllum nodosum* 4.5% of dry weight [6].

Laminarin could be utilized to get the activation of macrophages leading to immunostimulatory, antitumor and wound-healing activities, it is confirmed to have functional dietary fiber activity [141] and is a possible modulator of intestinal metabolism [143,144]. Laminarin decreases the levels of undesirable lipids such astotal cholesterol, free cholesterol, triglyceride and phospholipid in the liver. Additionally, it supplies protection against severe irradiation, decreases cholesterol levels in serum and reduces systolic blood pressure [69].

Laminarin, when ingested by animals, also acts as a dietary fiber [143]. Preparations containing 1→3:1→6-β-d-glucans, laminarin and fucoidan are manufactured by the health industry and commercialized because of their beneficial properties on the immune system [69].

Brown seaweeds also contain fucoidans in their cell walls. Fucoidans are a group of certain fucose-containing sulfated polysaccharides (FCSPs) representing the mixtures of structurally related polysaccharides with certain variations of monosaccharide residues and containing noncarbohydrate substituents (mainly sulfate and acetyl groups) [145]. Fucoidans are obtained from some species of brown algae, such as *Fucus vesiculosus*, *Sargassum aquifolium* (formerly *Sargassum binderi*) and *Saccharina japonica*. However, the chemical conformation of the majority of fucoidans is complex, composed of fucose and sulfate and other monosaccharides (mannose, galactose, glucose, xylose, etc.), uronic acids, acetyl groups and protein [146]. Additionally, the structures of fucoidans change from species to species in distinct brown algae [147].

Fucoidan can be used as an anticoagulant agent, as well as an antiviral agent and it exhibited antioxidant activity [148,149], having the potential to be used in the medicinal industry. It can also be used for skincare products (anti-cellulitis formulations) because it has moisturizing, anti-aging and anticellulite properties [150,151,152].

*Undaria pinnatifida* is rich in fucoidan used for skincare products (aromatherapy oil, face and body oil andbody scrub), which has anti-aging (anti-wrinkle), whitening/lightening, moisturizing and nourishing properties [2,153,154,155].

### 2.3. Red Seaweeds

The red algae (Rhodophyta) have their typical red coloration due to the pigments phycocyanin and phycoerythrin, in addition to chlorophyll [77,104]. Phycocyanin is the most important pigment in red seaweeds [156]. The commercial application of these compounds is as natural dyes, used nowadays in products, such as chewing gum, soft drinks, dairy products and cosmetic products, e.g., lipstick and eyeliner [157]. In addition, these compounds have health beneficial bioactivities, so they are indicated for nutraceutical products. Investigations have demonstrated their anti-oxidative, anti-inflammatory, anti-viral, anti-tumor, neuroprotective and hepatoprotective activities [158].

Inside the fatty acids, most commonly abundant SFA are myristic (C14:0) and palmitic (C16:0) acids [16]. The red seaweeds contain significant quantities of PUFA, mainly AA and EPA [159,160].

In red seaweeds, phenolic compounds asflavonoids and phlorotannins are abundant; having flavonoids three interconnected rings and phlorotannins above eight, doing more potent and stable antioxidants [161]. The phenolic compounds of red seaweed are being investigated for various industrial sectors, for example, pharmaceutical and cosmetic, due to their high antioxidant power [162,163].

For polysaccharides, red seaweeds produce agar and/or carrageenans. Agar is a linear polysaccharide composed of alternating (1,3) linked d-galactose and (1,4) linked 3,6-anhydro-l -galactose [164] and substituted in some degree by sulfate, methyl or pyruvate groups [91,165,166]. Agar has two main components: agarose and agaropectin.

Agar is found principally in the cell wall of the order Gelidiales (*Gelidium* and *Pterocladia*) and Gracilariales (*Gracilaria* and *Hydropuntia* spp.). *Agarophyton tenuistipitatum* (formerly *Gracilaria tenuistipitata*) is an economically important raw material for agar production due to its large number and easier exploitation [167]. The content and quality of agar depend on its specific physicochemical characteristics and are also closely related to environmental parameters [168], growth and reproductive cycle [169]. The best quality agar is removed from *Gelidium* spp. and *Pterocladiella* spp., while *Gracilaria* spp. yield low-quality agar [19]. *Gracilaria* spp. are one of the main producers of agar due to their fast growth and large agar content [170], being responsible for 80% of the global production of this phycocolloid [1].

The level of the algal protein content of *Gracilaria vermiculophylla* was increased by the accumulation of nitrogen (N) in the algal issue when cultivated in IMTA systems [171,172]. The quality and quantity of phycocolloid can change depending on the N content in the biomass [172,173,174]. The sulfated agarans from *G. corticata* have an antioxidant activity similar to well-known antioxidants (ascorbic acid and butylated hydroxyanisole, BHA) [175].

The main property of agar is the capacity to form reversible gels by cooling hot aqueous solutions. It is because of this ability that agar is used in many practical applications as a food additive or in microbiology, biochemistry or molecular biology in addition to other industrial applications [135].

Besides, agar oligosaccharides have biological activities such as antioxidant [176,177,178], antiviral [179], prebiotic [180], anti-tumoral, immunomodulatory, anti-inflammatory [176,181,182,183,184,185], inhibitory [176], anticariogenic [186], hepatoprotective [177] and other properties of interest for skincare [176,181,183,187]. *Gelidella* and *Gracilaria* spp. are extensively used not only for the production of agar but also for the treatment of gastrointestinal disorders [165].

For the pharmaceutical, cosmetics and food industries, agar is required as a gelling agent and stabilizing agent as well as a cryoprotectant [188,189,190,191,192].

Despite the low commercial exploitation of agar apart from the hydrocolloid industry, it has also been used in medicinal and pharmaceutical areas such as in therapy against cancer cells since it can induce the apoptosis of these cells in vitro [176].

The low-quality agar is employed in food products (frozen foods, bakery icings, meringues, dessert gels, candies and fruit juices) and industrial applications (paper sizing/coating, adhesives, textile printing/dyeing, castings, impressions, etc.) [169]. The medium quality agar is employed as the gel substrate in biological culture media as well as in the medical/pharmaceutical field as bulking agents, laxatives, suppositories, capsules, tablets and anticoagulants. The highest purified and upper market types (agarose) are employed for separation in molecular biology (electrophoresis, immunodiffusion and gel chromatography) [169].

In the last years, agar was also employed to develop a new biomaterial for packaging being sustainable, biodegradable and constituting an alternative to plastics [2].

Even if more than 90% of the world production of agar is employed in nutritional applications, significant commercial volumes are used in biotechnology [135] in applications such as electrophoresis, chromatography and DNA sequencing [193]. One of the most important applications is solid culture media for microbiology. The specific combination of features of several agars has made it the main gel former in this field. From *Gelidium* spp. are extracted the principal bacteriological agars and lower quantities from *Pterocladiella* spp. [135].

Carrageenans are high-molecular-weight linear hydrophilic, sulfated galactans formed by alternate units of d-galactose and 3,6-anhydrogalactose alternately linked by α-1,3 and β-1,4 glycosidic linkages [194]. Carrageenan is extracted from red seaweeds. Several groups of red algae show superior concentrations of one particular group and thus are known as carrageenophytes (carrageenan-producers), with most families belonging to the Gigartinales [195,196]. Several commercial red seaweed species supply a sub-family of carrageenan extracts.

Carrageenans are principally extracted from the genus *Chondrus*, *Eucheuma*, *Gigartina*, *Iridaea*, *Furcellaria* and *Hypnea* spp. Development and growing demand led to the introduction of the cultivation of *Kappaphycus alvarezii* and *Eucheuma denticulatum*, with a predominant content of κ- and ι-carrageenan, respectively, available all year [53].

Carrageenans have a backbone of galactose but are different in the percentage and location of ester sulfate groups and the proportion of 3,6-anhydrogalactose [135]. Kappa, iota and lambda (κ, ι and λ, respectively) are the most commercialized carrageenans, which can be independently supplied or as a well-defined mixture, due to the fact that most of the seaweeds contain hybrid carrageenans [197]. The bigger carrageenan yields can be over 70% (dry basis) for several species such as *Betapphycus gelatinum*, *Kappaphycus alvarezii* or *K. striatum*. Species such as *Eucheuma denticulatum* or *Chondrus crispus* have values near 30%. Sulfate content in carrageenans changes from 20% in κ-carrageenan to 33% in ι-carrageenan and 41% in λ-carrageenan [198].

The main source, *Chondrus crispus*, may be a model organism which includes a mix of κ- and λ-carrageenan [199]. *C. crispus* in its tetrasporophyte life phases produces λ-type carrageenan [193].

Recently, λ-carrageenan is highly promising for pharmaceutical and cosmetic industries. In the carrageenan food industry, cold-water species are unable to compete with the sub-tropical Asian carrageenophyte species. However, several authors are convinced that, in the medium to longterm, Asian carrageenophyte and agarophyte industries can go down because of climate change and the consequences in algal flora. Therefore, combining a cold-water carrageenophyte with novel market niches, such as cold-water *C. crispus* λ-carrageenan, can increase the feasibility of IntegratedMulti-Trophic Aquaculture (IMTA) [193].

Carrageenans are utilized in the pharmaceutical industry [198,200], focusing on anti-inflammatory [201], antiviral [202,203,204,205,206,207], anticoagulant [208,209], immunomodulatory [210], antitumoral [211], antioxidant [208,211,212], anti-angiogenic [213] and neuroprotective [211] activities. The function of carrageenans in agriculture has been verified [214,215,216,217,218,219,220,221]. They improve growth [222] and stimulate defense responses against viruses [214,221] and abiotic stresses [223].

Carrageenans are more employed than agar as emulsifiers/stabilizers in many foods, especially milk-based products. κ- and ι-carrageenans are specifically important for use in milk products such as milk, evaporated milk, ice cream, chocolate, puddings, jellies, jams, salad dressings, dessert gels, meat products and pet foods due to their thickening and suspension features [169].

Carrageenan produced by seaweed is not assimilated by the human body, acting as a fiber with no nutritional value, although it has a property that can be employed to gel, thicken and stabilize food products and food systems [135].

Around 70–80% of all carrageenan products are used in the food industry, which is still the main market for the algal hydrocolloids [224,225,226]. In processed meats, carrageenan is used as a water binding agent for preventing loss of moisture during cooking, increasing cooked yields and preventing an undesirable dry texture or bite. Carrageenan is used in toothpaste as a binder, similar to the carboxymethyl cellulose (CMC) [224].

Sauces, salad dressings and dips use carrageenan to give body, provide thickness and stabilize emulsions. The using of carrageenan has also been implanted in fluid dairy and dairy dessert products as the stabilization of cocoa, whipped creams and toppings [135].

Species of *Porphyra* spp. from red algae (Rhodophyta) contain a sulfated polysaccharide called porphyran, a complex galactan. Porphyrans are a family of agaroids polysaccharides produced by red seaweeds of the genera *Porphyra* and *Bangia.* They are composed of agarose highly substituted by *6-0-*sulfatation of the l -galactose units and 6-0-methylation of the d-galactose units [227,228].

Neopyropia yezoensis (formerly Porphyra yezoensis), Neopyropia tenera (formerly Porphyra tenera), Neopyropia haitanensis (formerly Porphyra haitanensis) and Phycocalidia suborbiculata (as Pyropia suborbiculata) are traditionally utilized in Japan as a food source. These algae are transformed into a sheet type of dried food, “Nori”, that contains main dietary fiber thatconstitutes around 40% of mass [229] and is famous in East and Southeast Asia, as well as globally, especially as a wrap for sushi [230]. Porphyran is a dietetic fiber of good quality and chemically resembles agar [69].

Porphyran exhibited significant antitumor properties against Meth-A fibrosarcoma. It can be perceptible lower the artificially enhanced level of hypertension and also blood cholesterol in rats [231]. Oligo-porphyran (acid hydrolysis product of porphyran) has the property to prevent and treat several pathologies such as Parkinson’s disease and acute renal failure [230].

In the releasing of histamine from the mast cells, porphyran is responsible due to its great inhibitory activity against hyaluronidase [229]. Additionally, porphyran serves as blood anticoagulant [232]. Porphyran’s relevant biological activities include anti-cancer [233,234,235], anti-hyperlipidemic [236,237,238], antioxidant [239,240] and anti-inflammatory effects [241,242,243] and/or immunomodulation [185,244] and avoidance of illness such as cardiovascular [63,87,236,238,245], nervous [246], bone [247] and diabetic disorders [248,249].

The modified Porphyran (with modified bioactivity and physical property) can be obtained by converting a salt of a sulfate group in Porphyran into a sulfate salt of a given salt by ion exchange. The modified Porphyran (with modified bioactivity and physical property) can be added and used in cosmetics, food, and drink, having inhibitory activity against hyaluronidase activity [250,251].

Porphyran is a gelling agent which provides gel strength in several formulations such as in toothpaste and is used as thickener and binder [252]. Additionally, porphyran has the function of stabilizing the tear film on the eyeball surface over a prolonged time and is used as an artificial tear liquid [252].

In the last years, some inventions have been made concerning the degradation of this compound to increase its utility in the pharmaceutical field, where, recently, porphyran has gained recognition [252].

## 3. Seaweed Aquaculture: Global Overview

**Figure 1:**
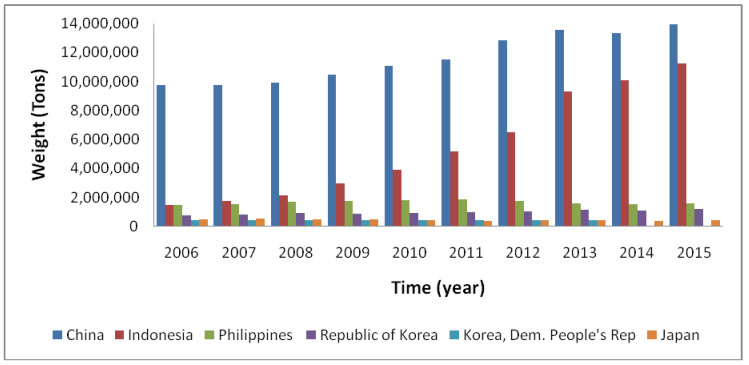
Global seaweed culture production by the main country producers, in tons. Adapted from FAO—The global status of seaweed production, trade and utilization, 2018 [1].

Over the past 70 years, seaweed farming technologies significantly developed in Asia, and, more recently, they have also gained position in the Americas and Europe [1,253] (see Figure 1).

There are historical registers of large-scale cultivation of seaweeds in Asia for decades [254]; however, in Europe and in other parts of the globe, this is a recent commercial activity [255,256].

The global annual production of seaweeds does not stop growing, reaching, in 2016, 31.2 million tons (fresh weight) [1]. Of this, just 3.5% was harvested from natural populations, in the time that 96.5% was produced in aquaculture, representing 27% of the worlds’ total aquaculture production [257]. The majority of this production happened in China, Indonesia and other Asian countries (47.9%, 38.7% and 12.8% of the worldwide production in 2016, respectively), mainly for human food and food additives [257]. The total aquaculture production of seaweeds exceeded more than the double in the last 20 years [1], and the total potential has been suggested to be 1000–100,000 million tons [258], but the main practice outside Asia is still to harvest natural stocks [57].

Besides the developments in seaweed aquaculture in countries such as China, Japan, Korea, Indonesia and the Philippines, there are also pilot-scale and pre-commercial farming projects for selected brown and red algae in Europe [259,260,261,262]; Latin America, for instance in Chile [263,264] and in Brazil [265]; the USA [266]; and parts of Africa [267].

Whereas the increasing global efforts to develop these farms, seaweed production and its commercialization strategies differ within the countries, as in the East there is a higher demand for edible seaweed as a direct food product, which produces higher incomes for farmers than the resources obtained from seaweeds’application in the polysaccharide industry in Western countries [268].

### 3.1. Environmental Requirements for Seaweed Aquaculture

The main environmental requirement for the seaweed cultivation is seawater with quality assessment without contamination. The seaweed needs to be native tothe location of aquaculture. Seaweed growth is always influenced by environmental conditions such as temperature, solar radiation, salinity, pH and nutrient availability [269,270,271]. Overall, to produce seaweed, areas with enough nutrients and light, as well as salinity and temperatures that do not limit the growing seaweed, are required [3]. However, distinct species of seaweeds need different environmental conditions [272]. Moreover, as life cycles are often complex, it is crucial to know the optimum or tolerable conditions to maximize seaweed production [273].

The main task in the seaweed aquaculture is balancing the positive and negative factors of the cultivation system to guarantee that the environment is not negatively affected or the status quo of the ecological system is not altered massively [272]. Thus, the seaweed aquaculture needs a detailed planning with various types of information (e.g., water quality, type of aquaculture, environmental pressure of the targeted location and socioeconomic impact), even before the targeted specie is chosen to assess and manage the risks to make decisions about how to minimize them and their negative impacts [272]. This is essential to promote a successful aquaculture in terms of production and ecological results.

### 3.2. Different Seaweed-Aquaculture Techniques

Seaweed cultivation can be performed offshore, onshore and even in aquaculture integrated systems. The culture of seaweed is chosen according to the species, place of the farm and cultivation facilities (see Table 1). In some cases, the techniques are identical, but the proportions are dissimilar due to the space restriction.

#### 3.2.1. Onshore Cultivation

Onshore or on-land cultivation started in the 1970s–1980s, trying to produce *Chondruscrispus* for carrageenan extraction [276]. This type of production takes place in closed systems (e.g., in tanks, raceways, ponds or lagoons) in which water is retained under agitation to keep seaweeds suspended and exposed to the light [277,278]. Tanks of different dimensions and numerous species can all be located together in one place, where they are easy to access, and specialized equipment is not required [277]. Possibly the main advantage of land-based cultivation is the monitoring and the opportunity of real-time adjustment of the conditions [268]. Inflows and outflows can be easily monitored, as seawater is pumped onshore and renewed depending on the cultivar needs. In addition, nutrients can be added efficiently, and therefore the composition of the media is under tight control [277,279]. Nutrient inputs can be precisely arranged to maximize the production of the bioactive compounds of interest while minimizing harmful discharge to the environment [268]. Furthermore, quality and quantity of light, as well as photoperiod, can be manipulated in order to achieve the farmer interests. Light quantity can be manipulated (by shading tanks or by handling the tank depth and seaweed density) and light quality can be controlled artificially by the use of greenhouse coverings and light sources [268]. There is also an easy control of pH and CO_2_.Giving that induced pH stress can be a useful tool for influencing real-time bioactivity content [280]. Salinity is also manipulated by mixing fresh/seawater ratios into the on-land tanks [268]. This leads to more consistent and standardized products obtained in these on-land systems, as there exists a better control over culture conditions [277]. However, seaweed densities can be handled, maximizing production levels in either fast-growing or slow-growing species [268].

Land-based cultivation methods have the advantage over ocean-based cultivation systems of adapting to a broad range of seaweed genera and forms, being suitable for all (except the largest) seaweed and allowing products to develop from non-dominant genus [277]. Farms are not so affected by adverse conditions such as tides, waves and wind. In addition, it is possible to produce small quantities of test biomass with high value for the market [277].

Main disadvantages of land-based cultivation are the high costs of infrastructure building and maintenance of farm conditions (for instance, in operatives work and energy). Land availability along with suitable water for land-based production is limited, andwhen available, it is normally expensive [277].

#### 3.2.2. Offshore Cultivation

The production of seaweed compounds for commercial products (such as polysaccharides) is not profitable in pond systems because of its high cost and so seaweed produced in these systems have limited their use to high-value products [268]. However, because of the lack of coastal space and its environmental impacts, and depending on the species of interest, an alternative to the land-based is the offshore production. It can be defined as a farm of marine products sited at a certain minimum distance from the coastline; however, it does not have a truly global legal meaning [281] and distance does not apply in many cases, principally when exposed conditions may be found within 1–2 km from shore [282]. Aquaculture in territorial waters should be legally considered as “coastal aquaculture” while aquaculture that takes place far beyond a nation’s territorial waters may legally be described as “Exclusive Economic Zone (EEZ) aquaculture” [283,284].

Naturally, seaweed growing and harvesting methods depend on the species. Seaweed offshore cultivation is practiced in adequate spaces, near shore areas including near farm concepts for kelp growth [285], tidal flat farms, floating cultivation [285], ring cultivation [286] and recently wind-farm integrated systems [287]. Harvesting techniques include hand-picking, cutting subtidal thalli to bulldoze andtractor or boat harvesting [288]. Skimmer boats harvest seaweed distant from the coast [33,289].

Seaweeds can either be produced on the sea floor (attached to hard substrate) or on long-lines (anchored lines or nets that are either seeded or have individuals tied to them for grow-out) [278,290]. Due to installation and maintenance cost being very low, attaching seaweeds to ropes, lines or nets is a popular way of cultivation [47]. The cultivation can be carried out attaching the seedlings straight away to the ropes [291] or via transplantation: seedlings are grown indoors, then cultured in greenhouse tanks for later the small fronds be transplanted onto ropes in the sea [292]. These cultivation methods are less costly and laborintensive for the maintenance of the seaweeds than the land-based ones [293]. Therefore, the offshore production of sustainable seaweed biomass is promising because of its sustainability, but extremely challenging at the same time [294,295].

In these farming systems, major issues are that the structures and seaweeds are susceptible to the most extreme effects of ocean and adverse environmental conditions. Farms have to be extensive and sited in numerous places to mitigate the environmental risk to the crop and to be economically viable [277]. Hence, there is the need to invest in structure design and materials that can last in rough sea states [47]. There are also additional costs and energy use involved in transporting the crops and operators to the farms [47]. Normally, these culture systems are placed in coastal waters which have strong water movement and are abundant in inorganic nutrient concentrations [296]. Seaweed cultivation can suffer fouling by macroscopic organisms such as bryozoans, epiphytic seaweed, hydroids, snails and blue mussels inducing deterioration of the algal tissue and causing high biomass loss [297]. The impact of such biofouling means that biomass must be harvested at late spring or early summer, limiting the cultivation period and the opportunities for accumulation of storage polysaccharides throughout the summer months [47]. Both the availability of nutrients in the ocean and the difficulty of doing the epiphyte control can be a problem, imposing constraints on seaweed aquaculture yield in these farms [47]. In this kind of cultivation, epiphyte over-growth is a considerable challenge [298,299]. For these reasons, seaweed species selected to open-water farming must be robust and resist epiphyte growth throughout the season to resist the local conditions. The adequate maritime area is currently not a limiting factor for expanding offshore agriculture [282], but climate change with the consequent changes in water temperature and water chemistry could lead to the reduction of suitable oceanic cultivation areas [300,301].

In the last years, offshore aquaculture has become an innovative research field due to the growing interest to move large scale aquaculture operations further out into the open ocean, demanding original solutions to tackle the challenges of the harsh and/or exposed environment [294,302,303,304,305,306,307,308,309]. Due to the hard-offshore environment, novel technologies for automatic cultivation and harvesting are required [47]. Normally, seaweed cultivation is, mainly, traditional and needs many unqualified, low paid hand labor [310] and intensified and automated cultivation may provide a new job sector that can collaborate to sustainable development in a lot of rural areas [47]. Thus, there is a need to develop a more robust and cost-efficient seaweed farming to withstand these problems [311]. However, various programs to develop the kelp offshore system have been conducted worldwide, in the last decade, with a small selection of cultivation systems surviving the harsh oceanic conditions, which improved the economic feasibility of offshore cultivation and can be critical solutions to surpass the problems that inhibit the development of this type of cultivation [311]. However, the results of the cultivation trials showed significant differences in the productivity related to the kelp species selected to cultivate and the farm structure design, becoming important to perform further work to ensure the durability and sustainability of these cultivation procedures [311].

#### 3.2.3. Nearshore Cultivation

The nearshore cultivation is the most known and used seaweed aquaculture technique, which can be developed in estuarine and near-coast locations [312].

This technique has the advantages of not competing for arable land (the problem of onshore aquaculture) and being protected by the land from mechanical aggression and damage provoked by sea agitation, sea storms and currents (the main problem of the offshore aquaculture) [313]. This technique has the advantage of facilitating the bioremediation of the river basins that are anthropogenically polluted with nutrients derived mainly from agriculture activity [313]. In addition, when compared to the inshore and onshore cultivation it is less costly and labor intensive [293]. This method is considered a derivative from the offshore cultivation technique, due to being inserted in aquatic systems [313]. Even though it is near the land, there is low interaction with it; the land is normally used as protection and land base for equipment. The mainly used cultivation techniques are line and net cultivation; however, there are general applications of on- and offshore techniques (as described in Table 1).

#### 3.2.4. IMTA Cultivation

Besides this simplistic insight on seaweed cultivation, as a singular production, seaweeds could even be combined in an integrated multitrophic aquaculture (IMTA) in order to solve some environmental issues of animal aquaculture, such as the eutrophication of the water because of feed supplementation and excretion [314,315]. The IMTA model is characterized by raising species from different trophic levels in proximity to one and other. Thus, the co-products (organic and inorganic wastes) of one cultured species are recycled to serve as nutritional input for others [104]. This type of cultivation brings benefits due to the interconnected cultivation: there is no need to add fertilizers to promote seaweeds growth, and the sustainability and profit are not in risk.

In IMTA systems, the animal’s (e.g., fish or mollusk) nutrient output, which is rich in dissolved ammonia and phosphate, is incorporated into the water, converting these compounds into a valuable biomass while stabilizing the levels of oxygen, pH and CO_2_ [47,104,172,313,316,317,318]_._ There are some studies about the effects of the effluents of fish production on the growth of seaweed. These investigations found that seaweed’s biomass increased when established in the fish farms [319,320,321]. Buschmann et al. demonstrated that a multitrophic culture of fed species together with seaweed as “extractive” species and filter feeders to absorb inorganic and organic nutrients, respectively, has the potential to reduce environmental nutrients from salmon aquaculture [320].

Species with higher productivity in summer, higher rates of nutrient uptake (hence, high growth rates), economic value andthat are easy to grow are previously identified as the most suitable for IMTA [294]. Through the development of appropriate models that can be used easily to locations anywhere in the world identifying suitable seaweed species and defining farm design to optimize the impact and economic return of IMTA will be aided hugely [322]. Thinning of crops is a normal farming method that optimizes growth by decreasing the limiting effects of self-shading. Kelp harvesting is used in practice too, by decreasing the length of the kelp and not by thinning of the plants [323]. A study was carried out to quantify the bioremediation potential of three seaweed species. One of the conclusions was that the height of the seaweed is a critical factor in its bioremediation potential. Different lengths were utilized to investigate how kelp optimizes its light environment and increases its nutrient capturing capacity in contrast to the smaller species that do not have the ability to grow over a large range of length. In a situation of light limitation, the seaweed could not grow until maximum biomass and its bioremediation capacity would have caught its upper limit [318,322,324].

Seaweed production, commercialization and utilization could contribute to other ecological bioremediation services, not only by ameliorating the water quality, but also the soil and atmospheric quality [29,57]. In fact, they could mitigate emissions from agriculture, by improving soil condition, substituting synthetic chemicals in agriculture by seaweed [29,325,326]. Investigations demonstrated the lowering of methane emissions from cattle when fed with seaweed [29,327].

The environmental benefits and positive externalities offered by seaweed aquaculture are bothlocal, such as reduction of eutrophication [328] and increased habitat for marine biodiversity, and global, by carbon sequestration and “blue” biofuel production [329,330]. In this perspective, to assist seaweed producers, rending their projects and companies economically viable, even in countries with highlabor costs, seaweed aquaculture should be taken into account to reduce its costs by subsidizing the algae aquaculture with environmental taxes [56].

Besides IMTA systems being able to reduce the ecological impacts of aquaculture, the production of algae can also bring financial benefits for the producers through the diversification of products they can commercialize and explore; gathering premium prices of the IMTA products. Marine seaweeds are ranked among the most efficient photosynthetic organisms on Earth and bear valuable chemical compounds [47]. Thus, IMTA also has advantages with faster production cycles [104].

Thus, seaweed cultivation on an industrial scale, particularly within an IMTA framework, can mitigate general pressure on the environment, as has been demonstrated in China [235,331]. In addition, offshore large-scale seaweed aquaculture may become a tool for carbon sequestration and to reduce global climate change [29]. Sustainability of aquaculture may grow through integrated cultivation systems [56,316,327,328,329,330,331,332,333,334]. Nevertheless, for IMTA to be economically profitable, all components must be marketable individually [335] or aggregate value to the ecosystem services that cultivated species provide [334].

#### 3.2.5. Saline Aquaculture

Another farming culture has become important in recent decades. In land, saline aquaculture is a land-based aquaculture utilizing saline groundwater. Saline lakes (ephemeral and permanent), saline water obtained with coal seam gas and saline groundwater extracted from aquifers are the sources of saline groundwater. Earthen or plastic-lined ponds, raceways and tanks (including those with recirculation mechanics) are several of the farming systems utilized [336]. Some of the advantages are that marine algae culture may use existing agricultural farms where saline water is available because it is less limited by the requirement for extra resource(s) and also because cultivating marine algae in island saline water (ISW) may supply an extra source of income and raw marine algae for the aquaculture seaweed industry, with a lower budget investment than farming in the sea [337].

Moreover, recent environmental studies have argued that diverse seaweed assemblages may have an advantage over monocultured seaweeds in the total nutrient uptake [320,338]. In short, researching in this field is mandatory, so seaweed would be even more studied, farmed and utilized. It is important to develop a comparison of bioeconomic models of seaweed sustainable production and to sensitize the population for this thematic.

### 3.3. Seaweeds Aquaculture in Major Cultivated Species

**Figure 2:**
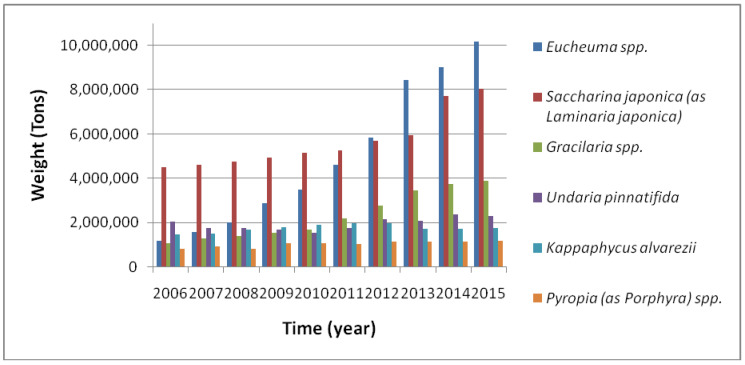
Global main seaweed species cultured, in tons. Adapted from FAO—The global status of seaweed production, trade and utilization, 2018 [1].

As the interest in seaweed-based products is increasing, the aquaculture of these species is growing. Currently, there are around 200 species of seaweed with a worldwide commercial use, of which about 10 genera are intensively cultivated, such as *Saccharina* and *Undaria* (brown algae); *Porphyra*, *Pyropia*, *Eucheuma*/*Kappaphycus* and *Gracilaria* (red algae); and *Monostroma* and *Enteromorpha*/*Ulva* (green algae) [253,339]. Figure 2 shows some of the most cultured seaweed worldwide such as *Eucheuma* spp., *Saccharina japonica* (formerly *Laminaria japonica*) and *Gracilaria* spp.

#### 3.3.1. Neopyropia/Pyropia

For example, for some species of seaweed, namely the species that reproduce sexually (e.g., kelps and the red algae *Porphyra*/*Pyropia* spp.), there are some specific requirements due to the complex life cycle. *Neopyropia*/*Pyropia*/*Porphyra* have been cultivated in Japan for hundreds of years and have become one of the major popular aquaculture industries in Japan, Korea and China [340,341]. *N. yezoensis*, *N*. *tenera* and *N*. *haitanensis* are the main species commercially cultivated (mainly in China, Korea and Japan) of the total of 138 species of *Neopyropia*, *Pyropia* and *Porphyra* accepted taxonomically [255,342]. During part of their life cycle (*Conchocelis* phase), they are produced in laboratories as support infrastructures. Thus, laboratorial conditions could be manipulated, depending on the intent of the producer, for maintaining seaweeds in a vegetative stage or shifting them to the next phase using temperature and light ranges, or even by tissue ablation [46]. Few farmers use free-living *Conchocelis* for seeding and others utilize *Conchocelis* on oyster shells [343,344]. Then, some of them must be attached and are, thus, restricted to the seafloor (benthos) or other substrate, such as strings [47,345].

For *Neopyropia*/*Pyropia* (formerly *Porphyra*) spp. to be successfully cultured, the seedlings when out planted need to be attached to a substratum, using a fixed pole, floating or semi-floating raft cultivation methods [46,346]. The epiphyte control techniques vary depending on the cultivation techniques. Many Chinese farms and some Korean and Japanese ones utilized desiccation control methods by leaving *Pyropia*/*Porphyra* nets to the air to take off epiphytes and competing organisms (e.g., *Ulva* spp.). Korean and Japanese farmers utilize an expensive pH control method by applying organic acids onto the nets [280,347]. Desiccation is a cheaper method which can increase the protein content in tissue. However, it is not as efficient as the pH control method [348].

#### 3.3.2. *Gelidium* spp. and *Pterocladia* spp.

Considering other Rhodophyta, such as the agarophytes, *Gelidium* spp. and *Pterocladia* spp., there are attempts to develop effective cultivation technologies. Although these algae can be cultivated in ponds and tanks, commercial cultivation is not generally considered economically viable due to their low growth rate [134,349]. Recently, two cultivation techniques have been tested: one involving the attachment of *Gelidium* fragments to concrete cylinders floating in the sea and the other involving free-floating pond cultivation technique. However, further investigations are needed since these cultivations are still not very successful and economically viable [132].

#### 3.3.3. Gracilaria/Gracilariopsis

*Gracilaria*/*Gracilariopsis* have been principally cultivated in China and Indonesia (70% and 28% of global production respectively) while in the Americas, Chile is the most productive country [255]. The majority of the biomass is utilized as the main source of food-grade agar [341] and as an animal feed [350,351]. Currently, 185 *Gracilaria* and 24 *Gracilariopsis* spp. are accepted taxonomically [342].

In contrast, *Gracilaria*/*Gracilariopsis* spp. are easy to propagate (asexually and sexually) and have relatively high growth rates [172,280,352,353,354,355]. They are euryhaline species, and, even though they tolerate a wide range of salinities (about 10–40 psu), they grow best in ranges of 25–33 psu [172,353,355,356,357]. They can endure temperature from 0 to 35 °C but have an optimal range of 20–28 °C [172,353,356,358]. These species have been successfully cultivated in open water rope cultivation, near shore bottom cultivation, pond culture and tank culture [341,346,359]. It could be necessary to have nursery cultures to provide sufficient seedstock through vegetative propagation [172,280,320]. The quality of wild *Gracilaria*/*Gracilariopsis* has been decreased because of the reduction in cultivation environments and the increase in diseases [360]. The use of asexually derived branches may lead to a decrease in genetic variability. Therefore, the development technology in hybridization, genetic material establishment while maintaining genetic diversity, will become very important [253]. *Gracilaria*/*Gracilariopsis* aquaculture challenge is to develop strategies and technologies to reduce fouling issues and identify solutions that may include freshwater rinses, utilization of tank growth fresh *Gracilaria* sp. seed stock, determination of optimal stocking density and photon fluence levels [253].

#### 3.3.4. *Kappaphycus* spp. and *Eucheuma* spp.

Other Rhodophyta, such as *Kappaphycus* spp. and *Eucheuma* spp., are cultivated using the same methodologies including the fixed, off-bottom line method, the floating raft method and basket method [341,361,362]. *Kappaphycus* sp. and *Eucheuma* sp. have been cultivated mainly in Indonesia followed by Philippines [255], being the major sources of carrageenan (over 80% of world’s carrageenan production) [341,362]. *Kappaphycus alvarezii* and *Eucheuma denticulatum* are the most frequently cultivated species where 6 and 30 species are taxonomically accepted, respectively, of each genus [253]. Site selection is one of the most important steps due to problems that organisms such as rabbitfish, turtles and long-spine sea urchins can cause to the farms [253]. Storm damage due to typhoons in tropical regions where cultivation occurs is also a problem. A solution to minimize storm damage is the removal of all cultivation systems before the typhoon season (~3 months per year). Development of more robust and cost-efficient farm systems are required specifically in the offshore environment. It is necessary to remove epiphytes 2–3 times every week, which needs intensive labor [363]. Thus, it is really important to develop novel strains that are light and thermally tolerant and disease resistant, as well as efficient epiphyte control [253].

#### 3.3.5. *Undaria* spp. and *Saccharina* spp.

Kelp has been used mostly for human consumption, but, in the last years, it has also been used as abalone feed due to the low production costs [364]. Consequently, over the last 50 years, kelp cultivation trials were being highly performed across the world to obtain the best cultivation method [311]. *Undaria* spp. and *Saccharina* spp. production has continuously increased due to demand for abalone feeds in Korea [364]. The kelp aquaculture industry in western countries has positioned itself as one of the fastest growing industries [50]. For kelps, such as *Undaria* spp. and *Saccharina* spp., cultivation starts with zoospores (meiospores) for seeding. The seeding methods differ between Asia (use of seed frames) and the West (use of seed pools) mainly due to the nursery capacities and the scale of operations of the offshore farms. After that, the offshore cultivation techniques using long lines are very similar [323,341]. The kelp thalli usually grow up to 2–5 m in length, although sometimes it may grow up to 10 m [323,341]. Due to selective breeding and intensive selection of kelp strains in Asia, there has been a reduction in genetic diversity and germplasm base of cultivated varieties [365,366,367], jeopardizing the expansion of the industry in Asia. In the United States, Canada and Europe, meiospores “seeds” have been primarily based on natural populations. The development of “seed banks” for algae species will provide a sustainable and reliable source of seeds without affecting the natural algae beds. Having seaweed with desirable morphological and physiological traits will also improve the production capacity of the algae industry [253]. Considering the cultivation techniques, kelps are the most suitable seaweeds to cultivate offshore due to their low requirement for maintenance and harvest in comparison to other species [253].

#### 3.3.6. Sargassum

*Sargassum* species have traditionally been used for food and medicine in Asia and continue to be wild harvested and cultivated in Japan, China and Korea [253]. In the beginning, traditional culture methods relied on the use of wild seedlings collected from natural beds (groups of 3–4 seedlings, 5–10 cm in length, were inserted into seeding rope at intervals of 5–10 cm). After, this seeding line was connected to a principal longline located at depths of 2–3 m and cultivated from November to May [368,369,370]. Due to this dependence on wild seedlings, there was an over-harvesting of natural beds and new culture methods were developed. Regarding *Sargassum* spp., the juvenile plants are obtained from reproductive adults. First, fertilized eggs are gathered from mature fronds. Then, they are “seeded” in lines, letting the newly forming rhizoids of the growing juveniles to attach to the line. These seedlings are cultured in a nursery tank until they are ready for out-planting at sea (offshore), in submerged long lines until harvest [152,323]. This is an economically feasible cultivation technique, but fouling organisms are problematic, so development to reduce fouling is an urgent need for the sustainability of the *Sargassum* aquaculture industry [253].

To ensure good conditions for the quality of the production, it is necessary to do the epiphyte control. There are different techniques that could be applied, depending on the species or cultivation methods [253]. For instance, one cost-less method for this is the desiccation, exposing the materials (the nets, lines) to the air to kill the fouling organisms. However, this is not as effective as the pH control that consists in applying organic acids onto the nets. This chemical control is also more expensive. The desiccation technique is used mostly in Chinese farms, while the Korean and Japanese farms prefer the pH control [280,347]. Regulating nitrogen concentration in the seawater of a land-based system is another method to perform epiphyte control [57].

## 4. Seaweed Aquaculture: The Aquaculture 4.0

Seaweed farming has developed as one of the alternatives to not exploit natural resources. At this moment, it is economically important in Asia and has a growing importance in Europe. The widespread potential of seaweeds application areas is comparable to other natural supplies such as palm oil and cocoa. Seaweeds are applied in product areas, such as cosmetic, medicine, biopolymers, food or even as a natural source to CO_2_ sink and biomass energy source [371]. The worldwide requisition to produce large amounts of seaweed will grow in the next years; however, until nowadays, there is still a continuous cultivation system optimization to deliver to this growing demand, a sustainable seaweed production and of their compounds [57,104,264,268,371].

However, collaborative work between academia and the aquaculture industry through research and development centers (R&D) has led to the development of research initiatives together to find new opportunities and new technologies to improve the efficiency and productivity in the seaweed aquaculture systems, making them more eco-sustainable and fit for the blue economy [277].

Camus et al. addressed some of the main problems that have an impact in the seaweed cultivation strain selection programs: the development of new massive plantlet production independent of collecting reproductive material every cycle; disease research; research on environmental impacts of large-scale cultures; and added value to the farmed species [264].

However, in Asia, the seaweed cultivation suffered a rapid evolution at the technological advances mainly in the floating raft cultivation systems, mainly for important species to the human consumption [341,372,373]. The major problem in the offshore aquaculture is the growth of juveniles in the sexual reproduction of selected species, for example kelps and *Porphyra*/*Pyropia* sp. This problem presents an expensive cost in the production chain, where the bigger scale can make this process affordable [374]. There is a need to develop reliable technology and cultivation strategies to achieve profitability [264,374,375]. Here, kelp cultivation is the most developed cultivation methodology system, due to the high interest in alginates and for human food [261,374].

There is a real need for the optimization of the current onshore seaweed cultivation techniques for the seaweed production [268,277,371]. The existing offshore cultivation system is not yet appropriate for setting out in deep-water or in the open water area, since the conceited aquaculture system is used in sheltered areas, and thus it is not possible to support more aggressive mechanical conditions. Consequently, the current onshore and offshore cultivation systems are not yet environmentally sustainable, and they are economically unstable, because the production fluctuates very rapidly, due to the impact of abiotic and biotic factors [57,261,371].

### 4.1. Seaweed Productivity and Quality: The Influence of the Abiotic Factors

The seaweed quality and productivity interdepend directly on the surrounding environment, which can be an advantage, because R&D and seaweed farmers can modify the abiotic factors to get a higher quantity of the targeted compounds of seaweeds than in the wild ones. This is one of the main advantages of onshore aquaculture. In addition, the possibility to cultivate seaweeds in more controlled environmental conditions and the cultivation of other species that are more difficult to cultivate in the offshore systems is another advantage [371]. However, until today in the onshore system, there is a general lack of available data on the hydrodynamic loading. These data are important to fully understand and optimize the onshore aquaculture systems. In addition, principal components analysis (PCA) correlating the seaweed productivity and the abiotic factors impact (in the aquaculture systems) is still needed to fully understand the aquaculture system. This will allow finding the best methodology to increase the quality of the cultivated seaweed. The abiotic factors will influence greatly the composition of the seaweeds, in different ways, such as light, salinity, pH, conductivity and nutrient concentration [268,376].

In general, there is a lack of data in the onshore/offshore aquaculture systems reporting the effects of abiotic factors in the productivity and quality of the seaweeds cultivated. However, there are studies presented in the literature for the wild populations of seaweeds, such as *Palmaria palmata* (Rhodophyta), *Ulva lactuca* (Chlorophyta), *Padina australis*, *Sargassum hemiphyllum* and *Fucus ceranoides* (Ochrophyta-Phaeophyceae) showing the correlation between the variations of compounds with the abiotic factors analyzed, such as UV radiation and salinity [318,376,377]. The output from aquaculture of water with high nutrient concentration can impact negatively the nearby ecosystem. Despite the high growth potential of seaweed (and high nutrient absorption rate), aquaculture reduces this problem from happening more frequently. However, this potential danger is one of the main problems, principally in the land-based cultivations [378].

Since 2018, studies are starting to emerge mainly in the integrated multitrophic aquaculture (IMTA) systems. For example, the study of Pliego-Cortés [379] correlated abiotic factors (stress tolerance and solar radiation) with the seaweed content of mycosporine-like amino acids, phenolics compounds and pigments. They provided data and a PCA analysis of the impact of the abiotic factors in the content variation in specific seaweed compounds. The study of Zepeda et al. [380] showed that the light quality will influence the red seaweed growth rate and pigment synthesis. Thus, it was possible to correlate the antioxidant activity with pigment concentration for different LED light treatment applications (light intensity). They concluded that more light intensity leads to a higher pigment biosynthesis. This type of defense response is related to the species’ ecology, as a survival mechanism under the environmental conditions of its wild habitat [380].

The studies presented by Pliego-Cortés [379] and Zepeda et al. [380] can be a reference to the aquaculture industry to get data and PCA analysis for the best location for specific aquaculture cultivations, as well asfor the pigment production. Additionally, seaweeds’ cultivation can be optimized to obtain a high pigment yield by controlling the light type, if artificial (low consuming LED with RGB systems linked), and its intensity or developing new techniques to control the solar radiation in the aquaculture system. This is greatly connected with the utilization of seaweeds in the industry in a wide spread of new applications of seaweeds’ compounds/extracts in various industries, which are becoming regular users of them [61,380].

Recent studies showed that it will be useful to fully understand the impact of the abiotic factors in seaweed species (wild or cultivated). Consequently, this knowledge can be very helpful to project new aquaculture systems to obtain higher quality from the targeted species, which is easier for onshore aquaculture than for offshore aquaculture, due to the variation and control of the abiotic factors.

### 4.2. New Multidisciplinary Analysis for Optimization of Seaweed Aquaculture

The seaweed cultivation process should be carefully analyzed from the very beginning of the cultivation planning. The planning for the cultivation location is very important to the target seaweed cultivation objective/production. Consequently, there is a need to obtain the maximum data of the targeted seaweed to understand every aspect of the aquaculture system. Thus, a new multidisciplinary level in the seaweed aquaculture is emerging, associating various types of engineering to enhance the seaweeds’ aquaculture to the next level: (i) computational fluid dynamics (CFD); (ii) mechanical and chemical engineering; (iii) informatics and electrotechnical engineering; and (iv) biological sciences and engineering. This new multidisciplinary approach applied to the seaweed aquaculture is very promising to improve the aquaculture/cultivation methods and techniques. This new era for seaweed aquaculture is developed under the denominated industry 4.0. Industry 4.0 is growing, suggesting the use of engineering and computer science coupled with multisensory schemes in the aquaculture systems associated with online servers and/or workstations, with logarithmic and artificial intelligence software to manage and control the system in every aspect and the change of different factors in the aquaculture system can provide a better aquaculture productivity and efficiency, reducing the overall costs [381].

#### 4.2.1. Computational Fluid Dynamics (CFD)

CFD deals with the simulation of systems through differential equations describing the most complete phenomena occurring in those systems. The aquaculture systems are based on the differential equations for the governing principles of fluid flow, heat and mass transfer, and in the (bio) chemical reactions. Those differential equations are then represented through algebraic equations which are solved numerically in time and space for the mesh elements [382]. For aquaculture systems, fluid flow is the principal target of the analysis and also the mass transfer and (bio) chemical reactions need to be considered in the model development. In addition as in the other CFD applications, for example in pulp flow [383] and microalgae production [384], the advantages of seaweed tank simulation over conventional experimental studies are the substantial reductions in lead times, experimental design and operation costs, and reduction of waste generated from experiments. Lastly, it is a powerful tool to carry out parametric studies for the aquaculture system optimization. Nevertheless, the CFD simulations and model development need to be validated through laboratory/or field studies for the most important cultivation factors [385]. In the end, the numerical results can help to defining a better design of experiments.

**Figure 3:**
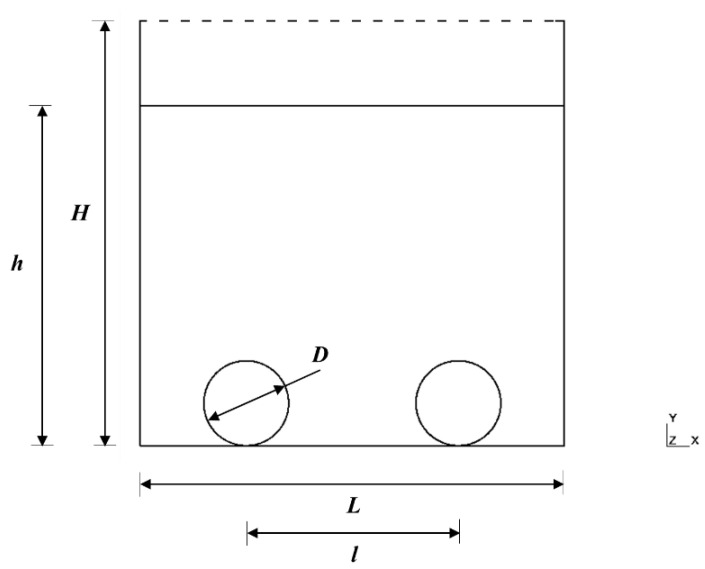
2D geometry of an aquaculture tank created in the open source software GMSH.

CFD simulations are very promising to obtain a good quality insight into the aquaculture hydrodynamics and the seaweed culture itself. Moreover, the biotic and abiotic factors that influence the seaweed environment can be taken into account in the CFD model to obtain excellent data to design a better system for the targeted seaweed/seaweed compound [386,387,388]. For example, it can be studied the tank geometry (see Figure 3), aeration flow and design aeration pipeline and, water recirculation through CFD simulations [386,387,388].

More complete CFD models include populations, showing the population as an entire group or a growth of a solo specimen and after that a scale up the effects to a population, to fully understand the impact in the cultivated seaweed of all the inputted variables in the model analyzed [279]. A minor number of super-individuals, which are the model individuals where each one symbolizes several hundreds or thousands of actual individuals, could also be introduced in the model, thus gaining a grade of in-population variability (individual based models). The usage of stochastic population models might also be a plan to decipher the deviation in progress on the individuals. The more advanced and sophisticated the model is, the further real growing data are necessary for “model training” and validation [279]. The advantage is the guarantee to have necessary data to improve the aquaculture to a next level of productivity and reduce the time in real cultivation trials.

Integrated multitrophic aquaculture with recirculation aquaculture systems (IMTA-RAS) is presently one of the best talented outlines of action to raise sustainability of fish farms and use seaweed cultivation to produce better systems. In the IMTA-RAS, either the bottom aeration or the impinging jet system, aimed to tumble seaweeds, symbolizes one of the main energy sink inland seaweed cultivation systems and their cost is a huge portion of the total production cost. Consequently, seaweeds’ movement and full tank hydrodynamics needs additional improvement to reduce the production costs [385].

CFD is one emerging area in the aquaculture systems that has a great potential to simulate these systems to contribute for a better knowledge on the system and to predict the proper geometry and conditions for a good seaweed production with lower energy costs than the current values. Overall, the CFD strategy applied to simulate aquaculture systems is an important tool to stimulate the aquaculture systems through the reduction of the time of assays, reduction of the production costs and the increase of the quantity and quality of the seaweed produced.

#### 4.2.2. Mechanical and Chemical Engineering

Mechanical engineering associated with the seaweed aquaculture provides the development of new materials and system designs to get more reliable materials and improve the processing technology for the aquaculture material, in onshore or offshore aquaculture. New technologies and the improvement of the existing ones are crucial to the seaweed industry and the development of seaweed cultivation. The technology in the seaweed production process is important to safeguard the seaweed aquaculture from any harm from the oceanic environment, which can inflict damage to the cultivation of seaweed. In the offshore aquaculture, along with the floating structural type farm, an efficient and trustworthy mooring system is essential to safeguard the seaweed aquaculture from any harm from the oceanic environment. [389]. Thus, the mechanical engineering studies are essential to create the most resistant aquaculture structure to survive storms and extreme abiotic factors, which influences the resilience of the seaweed aquaculture itself. The mechanical engineering field interlinked with CFD analysis can provide knowledge for better aquaculture structures, giving a reliable aquaculture system boosting the seaweed production and reducing the costs of maintenance.

Chemical engineering has a special emphasis in the measurements of the water quality, abiotic factors and seaweed quality. The data provided by chemical engineering represent important data input for CFD simulations. These data are also important parameters related with CFD simulation. The aquaculture location associated to the collection of the data are essential factor to obtain the most reliable data from the simulations to give the best tool for the decision making [390,391]. Thus, CFD models calibrated with experimental data represent a suitable and reliable support for the development of complete models to successfully characterize the aquaculture systems.

The chemical engineering also plays an important role to develop better materials to the aquaculture systems, associated with the mechanical engineering. In the end, both chemical and mechanical engineering can be useful to develop new materials more resistant for the aquaculture areas whether they are deep-sea, near shore or onshore.

These two areas present an important role and need to be interlinked to complete the knowledge, from each other and with CFD strategies.

#### 4.2.3. Informatics and Electrotechnics Engineering

The informatics and electrotechnical engineering field is gradually growing in importance to study the aquaculture systems. This growing interest is due to the general automation in the aquaculture systems, which allows the obtention of real-time data (RTD) to support the aquaculture management in order to help making decisions related to the complete aquaculture systems.

This field of engineering deals with sensors and data acquisition. In the multi-sensor technique, sensors are inserted in the aquaculture system to carryout real-time measurements of the quality of water and environmental parameters. These data can help to control the aquaculture systems in detail, helping the seaweed cultivation management to prevent seaweeds disease and contamination, the water quality or mechanical problems in the aquaculture. The main objective of using sensors is to control and maintain the quality and production of the cultivated seaweeds and reduce the cost of production through system optimization [381,392].

The multi-sensor technique has been proven to be very useful in aquaculture in the Yellow Sea, China by Xing et al. [392]. The multi-sensor remote sensing data from satellite and in-situ observations has been used to measure data of multi-temporal and spatial evolution in the *Neopyropia yezoensis* cultivation [392]. The objective of the study presented by Xing et al. [392] was the determination of the best location of the seaweed cultivation systems to prevent algal blooms that are harmful to *N. yezoensis* production in aquaculture.

#### 4.2.4. Biological Sciences and Engineering

This area combines seaweed biology, CFD simulations, material information from the mechanical engineering and measurements from chemical engineering. Moreover, this field completes the development of multidisciplinary models useful in assays either in the field or in the laboratory. Every real assay is done mainly by biologists working together with a multidisciplinary team of engineers. This fact is the principal link in the collaboration with the other areas to co-evolve the aquaculture systems to have better seaweed production and the reduction of the aquaculture costs.

The biology area is one of the most important because it is the area that knows the biology of the seaweed, and fully works in the field to understand the seaweed aquaculture. The other areas previously mentioned are important in order to help the evolution of the aquaculture to the next level, the aquaculture 4.0 (automation of the systems).

The study of Mantri et al. [393] is the perfect example of this new type of multidisciplinary study, where it is analyzed the aquaculture system from the beginning until the production using diverse types of tools to increase the production of *Hydropuntia edulis* (formerly *Gracilaria edulis*).

Azevedo et al. [394] studied theenvironmental factors ofthe classic vertical long lines with *Saccharina latissima*, which have been optimized during the years, and monitored the real abiotic data.

Another relevant study that presents data modeling is the study of the temperature modeling in onshore aquaculture system for the production of *Gracilaria pacifica* [395]. The authors created a CFD model solved in MATLAB that was associated with real-time data obtained from the aquaculture field assays. The results from the model revealed a great accuracy underneath most weather conditions in aquaculture location. In this work, they also made a series of possible modifications to the CFD initial model to give more strength in the production prediction. The model was strong enough to admit estimated values from various inputs and produce accurate results [395]. This study presented a model applied successfully to the prediction of production by day of the targeted seaweed. The model was validated through comparison of the simulation results with the data obtained in the field.

The seaweeds’ domestication to cultivate in aquaculture passes mainly by developing breeding and genetic improvement programs. The breeding programs are the most common, due to the seaweeds with a heteromorphic life cycle (such as kelps and *Neopyropia*/*Porphyra*/*Pyropia*) in which a multi-step cultivation technique is required. Here, the breeding is important for the success of the seaweeds first step in cultivation method, the obligatory sexual reproduction [396]. This breeding technique is very advanced and secure in the kelps and *Neopyropia*/*Porphyra*/*Pyropia* cultivation, due to the fully acknowledged seaweed life cycle and the longtime of these seaweeds’ domestication. In the isomorphic seaweeds, such as *Gracilaria* and *Kappaphycus*, the vegetative propagation is the reproduction technique used [396]. This last technique has higher survival rates and rapid growth, and the farmers can easily select the best phenotypes, unlike sexual reproduction of the large kelps [396,397].

In biological sciences, one last area has been explored and is gaining relevance to improve and develop strains that is the genomic area. This area tries to modify the genomic from seaweed to produce seaweeds with higher growth capability, better taste, darker color and higher resistance to diseases [398]. There was an exhaustive research on genetics, mutation, selective breeding and even genetic engineering in *Gracilaria*/*Gracilariopsis* spp. by Patwary and van der Meer [399]. The effects of climate change could be an encouragement to do more studies in the genetic manipulation area. Therefore, the technology development combining hybridization and genetic material establishment while maintaining genetic diversity can be very important [253]. However, the development and optimization of genetic tools in the seaweed world means that strains can be further developed and transformed, contributing to diversity. Nevertheless, the genetic modification of seaweeds is very restricted and faces many regulations due to the potential risks for human health and to the environment. Mainly, the genetically modified seaweeds are normally restricted in the closed aquaculture systems, not in the normal open cultivation methods [399,400,401]. This cultivation restriction also applies to non-native species due to putting the native species in danger and creating a very negative impact within the coastal ecosystem affected [57,396].

However, these genetic and breeding methods can create a reduction of the genetic variations within the species, which can be problematic to the species’ survival if the abiotic or biotic factors change [396].

### 4.3. Aquaculture 4.0: A New Era of Seaweed Cultivation

Industry 4.0 is related with the knowledge from engineering and computer science coupled with multisensory schemes for the aquaculture systems associated to online servers and/or workstations with the most appropriate software to manage and control the system, and, in this way, contributing for better aquaculture productivity and efficiency, reducing the overall costs [381]. Aquaculture 4.0 technologies are a sustainable alternative to optimize outputs (quantity and quality) and reducing costs and pollution in aquaculture [386]. Aquaculture 4.0 technologies and methods need to be developed to deal with the environmental requests from the aquaculture location and species cultivated; because the aquaculture can be offshore or onshore, abiotic and biotic factors influence the aquaculture system, which will have a high influence in the aquaculture productivity [279].

To obtain models for predicting seaweed production in aquaculture (e.g., the quantity of biomass that can be produced in specific systems), it is required to couple the seaweed growth models with CFD explicit models that provide the relevant environmental factors (e.g., temperature, nutrient concentration, photoperiod, carbon dioxide, light intensity andwater flow rate). This advance in aquaculture will involve multidisciplinary work to improve the traditional aquaculture systems with technologies and methods to optimize the seaweed cultivation, from the design phase to the harvest and processing of the seaweed biomass [279]. Aquaculture 4.0 scenario-testing tools can be used before the aquaculture is being created, for example, the “Modelling Approach to Resource economics decision-making in Ecoaquaculture”. This multidisciplinary tool gives the scenario insights at the economic and ecological level, critical for aquaculture sustainability. Consequently, all the economic, cultivation and socioeconomic data parameters can be analyzed to verify the dynamics of the aquaculture data to give a decision tool for the farmer. However, this tool can be further used with real data originated from aquaculture to help in making decisions about the evolution of aquaculture management [402].

The theory of Aquaculture 4.0 can be stretched to the seaweed farming management strategies that embrace the data gathering and exchange between joined nodes, to have real-time cloud computing practices and management [381]. More can be achieved in the aquaculture control systems, for example the Internet of Things, smart manufacturing, cloud computing and artificial intelligence [403].

Numerous technologies are presently in the implementation stage, in other areas that can be included in the Aquaculture 4.0: Recirculation Aquaculture Systems (RAS), offshore and onshore smart aquacultures, real time water quality [381].

The real-time monitoring of the water quality and aquaculture conditions developed by Aquaculture 4.0 programs are very valuable for farmers. These systems can give a great compact of information at intervals of seconds or minutes, making possible a more accurate planning of the aquaculture activities, and the possibility of prompting of alarms in case of unsafe water conditions/quality or weather alerts (e.g., permitting the offshore systems to descend the seaweed cage to deep sea weight, so the negative effect of the sea waves and the bad weather are lighter in the aquaculture system), also making possible analysis of instantaneous modifications in terms of time/or intensity in seaweed tanks. In addition, the production of a wide-ranging database that will help in detailed and specific studies in order to enhance the efficiency of the aquaculture in a medium and long time, lowering the risks and potentiating the seaweed aquaculture to another level (e.g., production of specific compounds).

The remote control and visualization of the RTD on a cloud-based platform is also one of the main advantages of aquaculture 4.0; mainly in marine farms, where the cages sometimes cannot be reached speedily at the wanted time. The cloud-based system of the onshore aquaculture parameters that can be accessed anywhere is also welcomed by onshore seaweed farmers [381].

In the European Union, there are mind-set and funding programs to evolve the aquaculture into the 4.0, being the breeding and aquaculture technology the major targets of the funding body, due to the critical key that can be crucial in the future for a more sustainable aquaculture [403].

## 5. Conclusions

The interest in seaweeds is increasing due to all the derived compounds and their bioactivities. They could have applications in nutraceutical and pharmaceutical products. There is an urge to produce and harvest more seaweed, in order to answer the higher demand of seaweeds and seaweed-based products. The scarce quantity of cultivated seaweed causes a real danger for wild seaweed populations due to the commercial over exploitation, causing huge marine environmental concerns. Thus, there is a need to provide more reliable aquaculture systems, in various formats (inland, nearshore andoffshore cultivations).

Several industries can use only one compound from seaweed and the science is evolving to understand how the seaweeds’ metabolism works, to obtain the best amount of the compound in aquaculture. Subsequently, the seaweed aquaculture technologies have been developed dramatically over the past 70 years mostly in Asia and more recently in the Americas and Europe. However, there are still countless challenges to surpass with reverence to the science and to social acceptability. In addition, in seaweed cultivation in various points of the world, it is still hard to use feasible and sustainable methodology and be economically and productive. The seaweed aquaculture has a long road for optimization.

The present main tasks in seaweed aquaculture embrace the development of disease resistance, fast growth seaweed species, high concentration of desired molecules methodologies and technologies, and the improvement of the aquaculture systems to be more robust and cost efficient that can resist storm events and maintain the cultivation during more time.

Progress in new cultivation technologies that can be more efficient and eco-friendlier is very important, so there is a need to have a multi and interdisciplinary team to optimize the aquaculture to the perfection to reduce the risks involved in the seaweed aquaculture and enhance new and better aquaculture systems and seaweed quality. In this way, the farmer can gain more control of the aquaculture systems. However, caution is needed to not over exploit the ecosystem sustainability, due to the danger of over-dosage of aquaculture with chemical fertilizers or other compounds that can and will reduce the water quality and damage the ecosystem, thus IMTA appears to be the best solution in terms of sustainability and profit.

## Figures and Tables

**Table 1:** Main techniques of seaweed cultivation. Adapted from Radulovich et al. [274]; and Sudhakar et al. [275].

	Onshore Methods	Offshore Methods
Line cultivation: -Off-bottom -Submerged hanging line -Floating line (long-line)	X	X
Net cultivation (depth, floating at the surface or slightly submerged)	X	X
Floating raft cultivation	X	X
Tank or pond cultivation	X	
Rock-based farming—direct planting on the ocean bottom or attached to artificial substrate		X
Onshore and offshore seaweed cultivation methods are identified by the c‘olor/shadow and a cross (X).		
